# Handling Public Well-being During the COVID-19 Crisis: Empirical Study With Representatives From Municipalities in Sweden

**DOI:** 10.2196/40669

**Published:** 2023-05-12

**Authors:** Ala Sarah Alaqra, Akhona C Khumalo

**Affiliations:** 1 Information Systems, Karlstad Business School, Karlstad University karlstad Sweden

**Keywords:** COVID-19, Sweden, government, well-being, public health, information and communications technology, recreational activities

## Abstract

**Background:**

COVID-19 has had a significant impact on the public’s health and well-being due to infections and restrictions imposed during the crisis. Recreational activities are important for the public’s well-being; however, the public’s safety from the COVID-19 virus is the top priority. Sweden, a country with a decentralized public health and welfare system, relied on less stringent approaches for handling the crisis. The limited restrictions in Sweden allowed recreational activities to take place despite the pandemic, which could be attributed to considerations for the public’s well-being.

**Objective:**

The objective of this study was to investigate municipal approaches for handling and supporting recreational activities during the COVID-19 crisis.

**Methods:**

We conducted an empirical study (qualitative and quantitative), using an online survey for data collection, with 23 participants. They were representatives holding mostly managerial roles in 18 distinct municipalities (18 counties). A thematic analysis was conducted to analyze open-ended responses, and descriptive statistics were used to summarize the closed-ended responses.

**Results:**

In this study, we report on the status of municipalities during the COVID-19 pandemic. The highlighted results showed a significant impact on the municipalities as a result of COVID-19, where 78% (18/23) of participants stated significant changes due to the pandemic. Moreover, 91% (21/23) indicated efforts and approaches for supporting recreational activities during COVID-19. Following national guidelines for the public’s health and safety was indicated by 78% (18/23) of participants. Information and communications technology (ICT) was considered significant for dealing with COVID-19 according to 87% (20/23) of participants. Our qualitative results further showed details of the public’s health and safety considerations, the efforts to support recreational activities particularly for youth, and the role and requirements of ICT. Challenges relating to the usability of ICT were also highlighted.

**Conclusions:**

Despite the critique of Sweden’s lenient strategy for handling the COVID-19 crisis, our results showed significant considerations for the public’s safety and well-being by the municipalities (regional and local levels) in this study. The Swedish approach to handling the crisis involved trusting the public with safety guidelines in addition to efforts for the public’s safety, supporting the public’s well-being with approaches for maintaining recreational activities, and giving special care to the youth. Despite having technological solutions in place, challenges in using digital solutions and requirements for future development were noted.

## Introduction

### Background

Sweden was recognized internationally for its loose response strategy in handling the COVID-19 pandemic. It received significant critique for not imposing stricter restrictions. This strategy resulted in high mortality rates and was labeled by news outlets and even expressed by the Swedish king as having “failed” to save lives [1], especially the lives of elderly people [2].

Response strategies for crisis handling differ depending on many contextual factors. Differences in institutional structures and cultural orientations are considered determinant factors affecting the response strategies among countries [3].

Sweden deployed the least stringent response for the COVID-19 pandemic, and the strategy was mild and advisory in nature, putting trust in individuals to take responsibility [4,5]. The contextual influence of this response is that Sweden is considered to have a loose culture (individual responsibility) and decentralized regime (authorities sharing power). In contrast, China, which is considered to have a tight culture and centralized regime, followed a stringent response involving authority-based coercive forces, which was drastic and mandatory, and wartime narratives were used [3].

The decentralized regime of the Swedish government includes a municipal local self-government for most public responsibilities [6]. Norway and Sweden are neighboring Scandinavian countries that share many similarities, including governmental and administrative systems; however, they differed in their strategies for handling the COVID-19 crisis. It has been reported that the rate of COVID-19–associated mortality was approximately 10 times higher in Sweden than in Norway (2.9 vs 0.3 per 100,000 person-weeks) [7]. Despite this, a study surveying individuals’ attitudes toward authorities and their compliance with restrictions reported that individuals in Sweden had a high level of trust in their government [8].

Recreational activities have been shown to be important for the subjective well-being of the public [9]. Studies have highlighted that enjoyable leisure activities can have physical and psychological benefits for health and well-being [10]. The COVID-19 crisis is a threat to public health, and well-being is affected indirectly due to restrictions.

The limited restrictions in Sweden allowed a degree of freedom for recreational activities to take place within the country, which could be attributed to consideration of the public’s well-being. We focus on the Swedish context in this study for its crisis handling strategy and its decentralized governmental structure (municipal responsibility) to investigate recreational activity support during the COVID-19 crisis.

### Objective of This Study

In our previous study, we investigated the impact of COVID-19 on businesses providing recreational activities, their status, and their coping measures during the crisis. The results showed trust in the government and the aiding role of information and communications technology (ICT) in coping with the crisis [11]. In this study, we follow-up with the governmental perspective, specifically the municipalities of the Swedish counties. Therefore, our objective was to investigate municipal approaches for handling and supporting recreational activities during the COVID-19 crisis. We targeted representatives with managerial roles from the corresponding municipalities to provide an account of municipal approaches.

### Theoretical Background and Related Work

#### COVID-19 and Well-being

When the COVID-19 pandemic hit, it swept through the world with terrible sickness and death. The cumulative death toll exceeded 6 million worldwide by June 16, 2022 [12]. Lockdowns and restrictions were introduced to enforce social distancing, an effort to reduce the rate of infection and preserve public health and well-being. Based on what was considered essential versus nonessential activities and services, more stringent measures were enforced in some areas [13]. In various countries, leisure activities were among those activities that faced closures and significant limitations, which had repercussions on mental well-being [14], emotional well-being [15], and physical well-being [16]. The negative effects on well-being are more pronounced when the reduction in leisure time for sports and exercise activities is involuntary, such as in the case of enforcement due to COVID-19 constraints [17]. Places of recreational and leisure activities that were affected during the pandemic included sports centers, gyms, swimming pools, museums, entertainment centers, theatres, hair salons, restaurants, spas, and other meeting places. The effects included financial impacts on organizations, impacts on the labor market, and impacts on the well-being of the consumers of the services provided.

#### Context of Sweden

Sweden has a decentralized public health and welfare system in which regions and municipalities have the right of rule in their jurisdiction; however, legislation is passed at the national level. When recommendations were issued, the government trusted citizens to yield to the recommended social distancing measures without coercion.

Variations among municipalities were seen between March 2020 and January 2021. Almost all indoor sports were closed, and then, when regulations changed, large parts of sports clubs were able to resume their activities. The restrictions at first required that there be no more than 500 people (March 12, 2020) in a single physical space. This was later reduced to 50 people (March 29, 2020) and then much later reduced to 8 people, impacting sport-related activities significantly [18]. The different measures were adjusted based on the rise or fall of the infection rate. Inevitably, the termination of sporting activities had a bearing on physical activity levels, especially among those who did not seek out alternatives.

The COVID-19 Act (SFS 2021:4, SFS 2021:8), a temporary act that came into effect in Sweden on January 10, 2021, introduced special limitations on certain activities and places to prevent the spread of COVID-19 [19]. However, after the COVID-19 Act came into effect, regions and municipalities could still make their own decisions on additional safety measures. For recreational areas, such as shops, indoor gyms, sports centers, and swimming pools, the Act imposed limitations on the number of visitors and distance between visitors, and imposed additional hygiene measures such as hand washing or hand sanitizing. The COVID-19 Act did not set limits on activities that could be carried out in an infection-safe manner [19]. Limitations placed on leisure and sporting activities did not apply to young people born in 2002 or later [20]. From April 28, 2021, children and young people born in 2002 or later were allowed to play outdoor matches once a week [21]. The majority of COVID-19 restrictions were lifted on February 9, 2022.

#### Sweden’s Administrative System

Sweden’s administrative system has the following 3 levels: national, regional, and local. The Riksdag, at the national level, passes legislation with execution supported by government offices. The 21 counties at the regional level, overseen by county administration boards, follow the Local Government Act, albeit with some autonomy. At the local level, there are 290 municipalities, which are also governed by the Local Government Act, with some degree of self-rule [22]. The councils at the regional level are mainly responsible for health care, while the municipalities are responsible for providing public services. Other national government agencies are also in place, such as the Public Health Agency of Sweden, which has the national responsibility for public health issues [23].

Because no hierarchical relationship exists between regions and municipalities, and each one is self-governed [24], municipalities within a region may differ in their approach and response to issues concerning public services, as each municipality has the right to make its own decisions. Even though Sweden has a decentralized health care system, national authoritative bodies, such as the Public Health Agency of Sweden (Folkhälsomyndigheten [FOHM]) and the National Board of Health and Welfare (NBHW), took more active roles during the pandemic [25].

#### Support for Recreational Activities

In Sweden, no restrictions were imposed on nature areas [26]. Being out in nature is beneficial in that it encourages physical activity, exposes the public to better air quality, fosters social cohesion, and reduces stress, all of which have a direct impact on physical and mental health [27]. Outdoor activities are also associated with positive youth development [28].

In a national study performed in Sweden [26], over 50% of participants increased their frequency of nature visits on weekdays and 45% increased their frequency of nature visits on weekends during the COVID-19 pandemic. Similarly, regional and local studies showed increased interest in and frequency of recreational activities outdoors in nature [26].

In Scandinavian countries, voluntary sports organizations play a big role in offering children and youth sporting activities [29,30]. Extracurricular activities afford children and youth opportunities to be in contact with peers and support systems outside the home, which they may need in times of distress [31]. In Sweden, sports activities that were managed by nonprofit organizations received financial support (provided to the sports and recreation sector by the government) during the COVID-19 pandemic [32].

Recreational activities are also essential for elderly people. Elderly people are often involved in nonvigorous physical activity [33]. However, they were more affected by restrictions on mobility due to COVID-19 as they were regarded as a more vulnerable group.

The population of Sweden is said to be highly physically active, which makes the country an interesting location to explore the effects of COVID-19 on recreational activities. About 70% of Sweden’s population is involved in regular physical activity [32], and a third of the population is registered with the Swedish Sports Confederation and its subsidiaries [32].

#### Digitalization and Digital Transformation

COVID-19 restrictions and responses to growing infection rates meant that many sectors could not operate or they had to change their processes. The need to continue operating encouraged the adoption and increased usage of digital alternatives, such as digital communication, online schooling, working from home, and making purchases online. Results from a study by Nagel [34] indicated that COVID-19 expedited the digital transformation of work. People who had the digital infrastructure and had occasionally worked remotely before the pandemic, for example, showed an increase in working remotely during the pandemic [34].

In some instances, the type of service offered was harder or even impossible to offer digitally. For others, the pandemic offered opportunities to reassess the possibilities digital offerings could bring to their companies and way of work. The leisure and recreation sector also made an effort to adapt to using digital technologies. In the case of endurance sports in Sweden, organizers adapted physical events into digital events, which allowed participants to use technologies, such as GPS and Strava, to record their activities and upload their results [32]. This meant participants could participate and go through the race as an individual. In these events, race participants could participate at the same time from different locations or at the same location at different times. The digital alternatives in endurance sports are promising, but participants seemed less than satisfied [32].

Other forms of digital offerings, such as online group exercises, which were offered through streaming services and facilitated online classes, were well received by both adults and adolescents in Australia during the times when in-person activities were not permitted, thus contributing to continued physical activity engagement [35]. Other forms of digital alternatives that saw a rise during the pandemic included telehealth, online games, online shops, and the use of exercise apps. Under the pandemic restrictions, people and organizations took up new habits and modified existing habits, including the use of digital technologies [36], and it remains interesting to see whether the new adoptions will be sustained after the pandemic. However, the exploration of digital alternatives also highlights the existence of digital inequality [37], though the inequality may not be too evident in technologically advanced countries.

## Methods

### Study Design

To achieve the objective of this study, we investigated the municipal approaches by exploring the perspectives and opinions of participants (representatives with relevant roles) working at the municipalities through empirical means. The empirical study was initially designed as semistructured interviews focusing on qualitative data. However, due to the pandemic’s circumstances, we adjusted and redesigned the study after the initial outreach to municipalities (phone and email correspondence). We converted our interview questions into an online format with survey questions. The online survey was both qualitative and quantitative, which consisted of closed-ended questions, several open-ended questions, and prompts for comments. There was a direct email correspondence with the study’s primary investigator for answering participants’ inquiries.

The online survey had 13 mandatory questions that included comments or open fields. The questions enquired about the following (see Multimedia Appendix 1 for the full survey questions): (1) The municipality which participants represent; (2) Participants’ position titles; (3) COVID-19 impact within the municipality; (4) COVID-19 impact on recreational activities; (5) ICT significance for dealing with restrictions; (6) Municipality approaches to aid recreational activities; (7) Municipality-specific restrictions for recreational activities; (8) Municipality measures to ensure safety of recreational businesses; (9) Recreational businesses not adhering to COVID-19 restrictions; (10) Municipality control for COVID-19 restrictions; (11) Municipality tracking of COVID-19 cases; (12) Newly adopted technologies/technologies used in new ways; and (13) Need for new technologies and purposes.

### Sampling and Recruitment

We recruited participants from the largest municipalities of the 21 counties of Sweden. We primarily reached our participants through the municipalities’ websites (in some cases, we used the region’s network). Online forms and chats on the municipalities’ websites, as well as email correspondence and phone calls were used to recruit eligible participants for this study. We mostly recruited those with “head of the department” or “leisure director” titles, and those having roles involving managerial or security responsibilities for the leisure activities at the municipality. We provided a brief introduction to the study and links to the surveys in both English and Swedish. We sent a reminder twice for participants who did not respond, and then, we expanded our municipality pool to include the second and third largest municipalities in the missing county. Eventually, our data covered 23 responses from 18 counties (from a total of 21 counties).

### Data Collection

We used a web-based survey tool named Survey and Report, which is supported by Karlstad University’s privacy policy. The estimated time to conduct the survey was approximately 7 minutes. Participants were provided with a consent form at the beginning of the survey (Multimedia Appendix 2), which included introductory text, details on the privacy policy and data management, and contact information of the study’s researchers. Data were collected after restrictions were imposed in late spring/early summer of 2021.

### Data Analysis

We used statistical descriptions as means to report on responses to the closed-ended questions (quantitative data). As for the open-ended responses, we analyzed qualitative data using thematic analysis. Thematic analysis is a method that involves coding the qualitative data followed by an iterative comparison of codes, which will eventually be categorized into themes [38,39]. The authors used the NVivo software tool (QSR International) to code, manage the merging of codes, and categorize the qualitative data from the study. The authors coded and formed themes separately. Between coding iterations, the authors met and discussed codes and interpretations of the results. Meetings for merging the codes, discussing the codebook, merging themes, and discussing conflicts took place. Themes resulting from the analysis are presented in the Results section.

### Ethical Considerations

We submitted an ethics application to our local ethics board and received the approval of the ethical advisor at Karlstad University for conducting this study. Participation in the study was voluntary, without any monetary compensation in exchange for participation; however, we offered to share the results of our study when published with our participants. We followed the General Data Protection Regulation (GDPR) and complied with the university’s policy for handling research data (details are provided in Multimedia Appendix 2). Participants were required to consent to the study in order to take part in it. They were anonymized, and no personal data were collected. Multimedia Appendix 2 includes the consent form for participating in the study.

In addition, the online survey tool we used is called Survey & Report, which is a web-based survey tool procured by Karlstad University. The tool is compliant with the university’s privacy policy and the GDPR.

## Results

### Participants and Overview of the Results

We present the results from the online survey, which includes both quantitative and qualitative data. We include the quantitative results from the closed-ended questions (statistical descriptions) together with the qualitative results from the open-ended fields (themes from our thematic analysis). In total, our study included 23 responses from 18 distinct municipalities. Gothenburg and Stockholm were represented by 3 participants each, Halmstad had 2 participants, and the rest had 1 participant each (Table 1). Most of our data are from the open-ended responses to the survey in the comments section for each question. Contributions varied among participants; however, only participant Mö did not contribute to the open-ended responses. All but 4 participants are in top management positions in the municipalities. The rest have the following positions: security coordinator (participant Kd), senior advisor (participant Ua), business controller (participant Sm3), and investigator (participant Sm2).

The results of the thematic analysis yielded 8 themes with corresponding subthemes (italicized), which are described in the following subsections. The statistical descriptions are present under the corresponding theme from the qualitative analysis.

**Table 1:** Overview of participant IDs and municipalities.

Number	Municipality	Participant ID
1	Göteborg	Gg1, Gg2, and Gg3
2	Halmstad	Hd1 and Hd2
3	Jönkoping	Jg
4	Kalmar	Kr
5	Karlskrona	Ka
6	Karlstad	Kd
7	Linkoping	Lg
8	Luleå	Lå
9	Malmö	Mö
10	Nyköping	Ng
11	Stockholm	Sm1, Sm2, and Sm3
12	Sundsvall	Sl
13	Umeå	Uå
14	Uppsala	Ua
15	Västerås	Vs
16	Växjö	Vö
17	Östersund	Öd
18	Örnsköldsvik	Ök

### Impact and Changes in the Municipal Working Environment

The majority of participants (18/23, 78%) stated that their municipalities experienced significant changes in communication, work processes, and service provision since the start of the COVID-19 pandemic. Three participants’ municipalities (3/23, 13%) had undergone few or minor changes, 1 participant’s municipality (1/23, 4%) had undergone major or critical changes, and 1 participant’s municipality (1/23, 4.3%) had undergone no changes at all.

The subtheme of *municipal compliance with regulations* included following infection control guidelines, the COVID-19 Act, recommendations by the government, and the FOHM.

It was noted that there were *municipality-specific regulations, adjustments, and restrictions*. It was indicated that municipalities followed regional rules (participants Hd2 and Ua), local rules (participants Hd1 and Lg), and additional restrictions and constraints (participants Uå and Ök). Participant Ök stated:

For certain activities, the national guidelines apply, but for open leisure activities, the guidelines that are prepared locally for measures that aim to meet the risk assessments made for each activity apply.

In addition to restrictions, the impact of COVID-19 on the municipal working environment resulted in changes in communication (participants Hd1, Lå, and Sm3) and logistics (participants Sm1 and Sm3). Participant Lå stated:

Very many of the changes are related to communication with both the general public and the association life.

Moreover, participant Hd1 mentioned:

In addition to the administration's officials mostly working from home and all meetings take place in digital form.

The impact of COVID-19 restrictions on staff members resulted in digital and remote work (participants Gg1, Gg3, Hd1, Kr, Sm3, and Uå), change in staff responsibilities (participants Hd1, Uå, and Ök), working from home (participants Hd1 and Kr), change in the work environment and spread of staff (participant Uå), and significant work to enable activities following the recommendations (participant Sm3). Participant Uå also stated that the municipality collaborates with trade unions for allocating staff.

### Efforts Supporting Recreational Activities

Responses from the closed-ended questions showed that 91% (21/23) of participants had some approaches in place to aid recreational activities during COVID-19, such as keeping recreational activities ongoing or starting new outdoor public activities.

From the thematic analysis, efforts supporting recreational activities included *allowed activities within the scope of restrictions.* Several responses from 10 municipalities (participants Gg3, Hd1, Kd, Kr, Lg, Ng, Sm1, Sm3, Uå, Vö, and Ök) indicated that they had approaches to aid recreational activities, and participant Kd stated that activities were based on FOHM recommendations. One approach was the *provision of alternatives*, which included alternative premises (participant Gg3) and outdoor activities (participants Gg1, Gg3, and Vö). Participant Gg3 indicated that in the municipality, staff members “review alternative methods to be able to support the pursuit of leisure activities.” Efforts for *enticing open activities* included local measures for open leisure activities (participant Ök) and advertisement of open areas for activities (participants Hd1 and Sm3). Participant Hd1 stated:

We have marketed activity areas, playgrounds, nature areas, and outdoor sports fields in a better and more targeted way to encourage spontaneous sports.

Participant Lg also indicated extra support for sports and cultural activities, and stated:

Several decisions have been made to support the pursuit of leisure activities, such as extra support for sports and culture.

However, participant Sm3 indicated challenges to youth support despite the alternatives provided, which included the challenge of student support and encouraging youth outdoor activities. Participants indicated *support for association life* or “föreningslivet” in the form of communicating rules (participants Lå and Uå), facilitating logistics (eg, easier bookings; participant Uå), communicating information about restrictions (participant Uå), finding creative ways to support sports activities (participant Hd1), and financial support (participants Kr, Sm1, and Uå). Participant Kr stated that in 2020 “SEK 10 million was paid out in extra association support.” A currency exchange rate of 1 SEK=0.1 USD is applicable.

### Youth-Focused Support of Leisure Activities

Support for leisure activities with a focus on youth has been noted in our results. Given the restrictions imposed by the government and recommendations by the FOHM (health authority), participants from municipalities indicated that special measures were taken, especially for youth and children (participants Hd1, Kr, Ng, and Sm3). Support for youth was in the form of *access to activities*, which has been granted exclusively for youth and children (participant Kr), and holiday, cultural, and sports events and activities targeting youth (participant Sm3). There were also efforts to keep leisure activities for youth (participant Ng), and participant Ng stated:

We have tried to help keep as many leisure activities for children and young people as possible.

Participants also indicated their *provisions of places for youth activities*, where they provided physical meeting places for youth as an alternative to digital meetings (participant Hd1), and kept preschools and park games open (participant Sm3).

Participants Sm3 and Hd1 highlighted the importance of leisure activities and places as a safe environment for youth. Participant Sm3 stressed the following:

Recreation centers historically reach boys and girls living in various forms of vulnerability (economic or social) and are a safe place for those who need it most, which is why a total closure of the activities potentially has far-reaching consequences.

Participant Sm3 added that extra seasonal staff were employed to expand on existing activities in “park games (target group 7-12 years) and in leisure centers (target group 12-19 years).” However, the challenge of “complying with restrictions and at the same time carrying out the core mission of creating safe places, context, and meaningful leisure time for children and young people” (participant Sm3) was also indicated.

### Impact of COVID-19 on Recreational Activities

In general, there were *limitations to recreational activities due to COVID-19.* Participants from 9 municipalities indicated that due to the recommendations from authorities, recreational activities were closed (participants Kd, Kr, Hd1, Hd2, Lg, Lå, Sm3, Ua, Uå, and Vö). For example, swimming halls were closed to the public (6 municipalities; participants Hd1, Kd, Kr, Lå, Sm3, and Uå). Participants from 7 municipalities indicated limitations and constraints to recreational activities (participants Gg3, Hd2, Kr, Lå, Sm1, Ua, and Uå), whereas participants Ka, Jg, and Lå indicated a decrease in recreational activities. Participants from 5 municipalities noted a decrease in the number of visitors for recreational activities (participants Hd2, Ng, Sl, Vs, and Öd). Public health was a concern as a result of closures, especially for youth and associations, as indicated by participant Uå:

Closure of sports, leisure centers, bathhouses affects public health, loss of active 7-20 years in association life for 2/3 of the associations.

COVID-19 restrictions had an impact on the operation of recreational activities. Some reported complete closure (participants Sm3, Uå, and Ök), while others reported closure for cultural activities (participant Hd1), leisure and sports (participant Uå), gyms (participant Sm3), some periods (participant Lg), and libraries (participant Sm3), or general closure and limitation of activities (participants Hd2, Ua, and Vö).

However, *exceptions to the restrictions of activities* were noted. As some activities were operational for associations (participants Hd1 and Öd), priority was given to the activities of young people (participants Ua and Sm3), and exceptions were made for prescription and rehab visitors (participant Sm3).

### Public Health and Safety

To ensure safety in recreational businesses during the pandemic, among the 23 participants, 18 (78%) said their municipalities followed national guidelines and 5 (22%) indicated that their municipalities followed municipality-specific guidelines. Additionally, 11 participants (48%) said municipality-specific restrictions for recreational activities were put in place in their municipalities, 11 participants (48%) indicated that their municipalities did not have municipality-specific restrictions, and 1 participant (4%) did not know.

Participants highlighted the importance of the public’s health and safety regarding COVID-19, where they indicated *efforts to ensure safety* and *efforts to comply with restrictions*. Their efforts included the following: general guidelines (participants Hd1, Kr, Lg, Lå, Sm1, Ua, Uå, and Ök), national guidelines for certain activities (participants Hd1, Kr, Lg, Lå, Ua, and Ök), and municipal guidelines (participants Sm1 and Uå). Participant Lg specified following the FOHM advice and the COVID-19 Act. Participant Ök mentioned conducting risk assessments for each activity. In addition, participant Hd1 stated that there were stricter restrictions in the Halland region, and participants Uå and Ua indicated that local decisions were followed depending on the situation. Participant Ua stated:

The starting point in the municipality's work is national guidelines. Due to the epidemiological situation in the region or municipality, adaptation has sometimes had to take place, this is done in consultation with a regional infection control doctor.

To limit the spread of the infection, participants indicated that municipalities had further efforts to *limit contact* for recreational activities (participants Kr, Lg, Lå, Sm1, and Ua). Participant Lå stated:

We apply the general guidelines to limit the number of people who can stay in the larger facilities at the same time, as the COVID-19 Act allows too many in the same place at the same time.

The mentioned efforts included keeping unnecessary activities closed (participant Lg), applying rules to limit the number of people at bigger facilities (participant Lå), and closing all public activities at peak infection times (participant Sm1). Trust in the public was noted, as participant Gg1 pointed out that they expected visitors who were infected with COVID-19 not to visit their facilities and spread the infection.

Regarding responsibility, it was indicated that there was a *distributed responsibility for monitoring adherence to restrictions.* Among the 23 participants, 17 (74%) indicated that their municipalities controlled adherence to COVID-19 restrictions, 2 (9%) indicated that their municipalities did not control adherence, and 4 (17%) did not know. A small number (3/23, 13%) of participants indicated that there were recreational businesses that failed to adhere to COVID-19 restrictions, 8 (35%) cited that there were no instances of nonadherence, and 12 (52%) did not know.

Some participants indicated that municipalities controlled their own activities (participants Kr, Kd, Hd1, Hd2, Sm1, and Uå). Participant Hd1 stated:

The activities for which the municipality is responsible (eg, meeting places in culture, swimming school, library) follow the restrictions closely.

Moreover, participant Uå stated:

Own municipal activities are monitored daily. We support association life but do not take responsibility for their own routines as they have the same responsibility.

Associations were expected to have control over their own activities (participants Hd1, Hd2, and Uå). Participant Hd1 explained as follows:

Since it has been up to the associations to ensure that their activities follow the restrictions, we cannot guarantee that it is done, based on the fact that we are not always in place.

However, some participants indicated periodic checks by the municipality to monitor activities in general (participants Hd1, Kd, Kr, Sm2, Sm3, Lå, Ng, Ua, and Öd) in the form of check-up visits but without control (participant Hd1), informing related associations in case of violations (participant Lå), and random sampling of activities (participants Kr, Ng, and Öd). Some indicated that it is still unclear where the responsibility lies (participants Kr, Gg2, Sm1).

Moreover, *measures to track COVID-19 infections* were noted. Among the 23 participants, 14 (60.9%) reported having no measures in place for tracking COVID-19 cases in their municipalities and 9 (39.1%) reported having tracking in place within their municipalities. Participant Kr stated:

The municipality participates in infection tracing when necessary and closely monitors the development of the activities.

Participants indicated that they traced infections in their municipalities (participants Kr, Gg2, Sm3, and Ua), followed routines by infection control (participants Sm2 and Uå), and performed infection tracing among staff (participant Kr). Participant Gg2 explained about infection tracing as follows:

Infection tracing about an outbreak, for example at a meeting place.

In addition, participant Sm1 indicated that this was done as a response to requests by Infection Control (Smittskydd).

Participants also indicated that there were *issues following restrictions*. Participant Uå explained as follows:

We do our best to comply with the restrictions, which I understand we do to 99%. We consult with infection control in (our region) and within our municipality. Sometimes it is difficult to interpret the restrictions as a municipality.

The issues mentioned were as follows: not all individuals followed the guidelines and restrictions (participant Sm3), restrictions were not taken seriously (participant Hd1), and individuals were not fully adhering to restrictions (participant Öd). However, municipalities did not monitor nonadherence to restrictions, as participant Hd1 mentioned that they were not always around the premises to witness and could not guarantee adherence to restrictions. Participant Lå stated:

We do not carry out follow-up as we do not have a mandate for this.

According to participant Sm3, there were challenges following restrictions for youth and children. They indicated difficulties conducting leisure activities given the limitations, and there were issues maintaining social distance during activities for children and youth. Furthermore, staff faced challenges with ensuring children’s health in a public environment given the restrictions in place.

### Role and Significance of ICT

We inquired about the role of ICT within the municipalities during COVID-19. Among the 23 participants, 20 (87%) highlighted that ICT played a very significant role in dealing with COVID-19 restrictions in the municipality, 2 (9%) stated that ICT was somewhat significant, and 1 (4%) remained neutral. Additionally, 21 participants (91%) stated that they adopted new technologies or were using technologies in new ways.

In the open-field answers, participants mentioned the ways technologies were used. *Digitalization of processes* was prominent in their responses (participants Hd1, Kr, Lg, Lå, and Uå). Some examples are digital meeting tool acquisition (participant Hd1), immediate implementation of cloud applications (eg, Office 365; participant Kr), development of digital methods (participant Hd1), regular digital meetings with associations (participant Lå), and expansion of technical solutions (participant Lå). Participants stated the increase of digitalization and use of ICT (participants Gg2, Gg3, Kd, Ng, Sm1, Sm3, and Ua), intensive use for remote work (participants Sm2, Sm3, and Ua), use of social media and digital communications (participants Ng, Sm1, and Sm3), and use of new digital solutions for recreational activities (participants Ua and Ök). Examples of new digital solutions used for activities included a new platform for remote collaboration (participant Lg), new digital solutions for library and youth activities (participant Ök), and new online solutions for youth games and quizzes (participant Gg3).

Furthermore, participants mentioned the *significance of ICT in enabling communication and maintaining connections* within the organization and with the public. Acknowledging the importance of ICT was noted (participants Kd, Hd1, and Sm3). Participant Kd stated:

The situation requires a lot of digital meetings, which is why communication technology is important. In terms of communication to citizens, we feel that digital solutions are less important for reaching the public.

Participant Hd1 added the importance of maintaining updates and routine communication (using ICT) with the public to provide reassurances as follows:

Perhaps the most important thing is not what you communicate but that you communicate. Even if you send an email with content that says, “I have no new information to provide” or “there will be no changes,” it creates calm and confidence for our residents and visitors.

### Challenges in Using ICT

Participants highlighted *challenges in using ICT*. Participant Gg1 highlighted ICT security and privacy issues as follows:

The city has not developed so that we can meet the target group digitally. Unsafe platforms that the municipality does not approve. Difficult with GDPR. Difficult with digital meetings with the target group.

Different barriers to acquiring new ICTs were also highlighted, such as competencies, resources, technicalities, and licenses. Participant Gg1 stated:

There are some unresolved issues regarding permits and licenses that could open up further.

Moreover, participant Ök stated:

There are many different technical solutions, but it can be a matter of both skills and money to be able to use the solutions.

Participants also mentioned *usability issues with digital tools,* being unfamiliar with the tools (participant Gg1), and inability to communicate what the recipients want (participant Hd1). Furthermore, the limitations of ICTs were highlighted as they are not to replace physical communications (participants Kd and Sm3).

### Future ICT Needs and Requirements

Participants from 12 municipalities (55%) indicated that there is a need for new ICTs to address various requirements and needs (Table 2).

Additionally, the need for inclusive ICTs for elderly, youth, and disabled people was mentioned by participants (participants Hd2, Sm2, and Uå). Digital isolation (a concern that disabled people are not able to participate in digital group conversations) was highlighted as a concern with nonsuitable digital tools (participant Sm2). Furthermore, the need for a simpler and usable technology for the target group was highlighted by participant Sm2 as follows:

Development of “easier” aids and communication paths with fewer steps, today the technical readings are often too difficult as connecting, adjusting sound and clicking in and out to avoid rounding, need to be done in too many steps for e.g. elderly. This discourages and puts the target group at risk of digital isolation.

In addition, participants Uå and Hd2 specified the need for technologies targeting youth and young people, by either providing digital meeting spaces or providing better services (eg, borrowing techniques).

**Table 2:** Requirements for information and communications technology indicated by participants.

Requirement ID	ICT^a^ requirement description	Participant ID
RQ1	Development for communication, dissemination, and meetings	Lg and Hd2
RQ2	Enhancement of the efficiency of running businesses	Jg
RQ3	Development for connectivity	Hd2
RQ4	Development of better technologies	Öd
RQ5	Strategic business operations	Sm3
RQ6	Creative business processes	Sm3
RQ7	Improvement of services	Jg
RQ8	Value creation for visitors	Sm3
RQ9	Development of the interactivity of digital activities	Sm3

^a^ICT: information and communications technology.

## Discussion

### Summary and Overview

By targeting a municipality in each county, we attempted to obtain the views of all counties in Sweden, thereby gaining insights on the approaches of municipalities for handling and supporting recreational activities. Due to the positions held by the participants, it can be assumed that they are well-informed and speak from a position of authority. By this we mean that they are likely to have a clear understanding of how their municipality works and are well-informed about municipal decisions and operations during the pandemic.

According to our results, most of the participants have seen significant and major changes in communication and working processes (18/23, 78% and 1/23, 4%, respectively) within their municipalities, have an increased focus on ensuring public health and safety (18/23, 78%), continue to adopt means to provide recreational activities during the pandemic (21/23, 91%), and have seen an increase in the use of technology (20/23, 87%). A strong consideration for youth well-being and high regard for complying with public health guidelines were seen.

COVID-19 impacts municipalities in various ways. To ensure public and employee health and safety, municipalities needed to incorporate changes in their processes to ensure compliance with regulations, implement municipality-specific regulations and adjustments, and address the impact and changes to the municipal working environment. We discuss these impacts and the 8 themes highlighted in our results in the following sections.

### Public Health and Safety

Though municipalities in Sweden are at liberty to make their own decisions, the responses indicate that municipalities prioritize following national guidelines, consult with their respective regions, and have added municipality-specific regulations and adjustments as necessary. According to participants, the national guidelines followed were those issued by the FOHM. With the responsibility for health care, it could be expected that regional councils would have an interest in the measures being implemented within the region, as well as the level of stringency. For the regions, a wide spread of the virus would put a huge strain on health care resources. Thus, curtailing the spread of infections would be paramount. It must be noted that the COVID-19 Act only came into effect at the beginning of 2021, enforcing specific closures and limitations based on numbers and spaces. Before the Act took effect, various measures were already introduced by different municipalities to a higher or lower degree, and even following the Act being in place, some regions and municipalities believed the restrictions were not stringent enough. As highlighted by 1 participant, the measures that apply to open leisure activities are decided by the municipality following a risk assessment specific to each activity. Some municipalities made efforts to further reduce the number of people allowed by the COVID-19 Act in larger facilities. In addition, municipalities decided to close activities deemed unnecessary or at peak infection times. The measures implemented by municipalities indicate a keen interest in maintaining public safety that goes beyond meeting minimum requirements. The lack of strict enforcement from the government at the national level does not necessarily translate to leniency toward protecting public safety at the local level. The public is also trusted to act responsibly and keep away from facilities when infected.

Responsibility for monitoring and ensuring adherence to regulations was distributed and did not solely fall under municipalities. Over 70% of the participants (18/23, 78%) stated that municipalities ensured that safety measures pertaining to them were implemented, but did not have the responsibility to ensure adherence by associations as associations had the same responsibility toward themselves and their activities, with a level of autonomy between the two. Municipalities controlled and monitored activities for which they were responsible, to ensure adherence to safety measures, and they could not guarantee adherence by associations. However, municipalities could randomly sample activities and perform periodic checks, and could inform the relevant association in the event of a violation. A concern could arise where municipalities are uncertain of where the responsibility of ensuring adherence to regulations lies, as this would suggest a gap in which the responsibility is possibly not being fulfilled. Though municipalities did not control the activities of associations or the adherence of associations to restrictions, participants communicated that they offered support to associations during the COVID-19 pandemic. The forms of support ranged from sharing information regarding the pandemic and its related regulations and restrictions, as well as sharing creative alternatives, to supporting sports activities and facilitating smoother processes for accessing financial support. A comment highlighting that SEK 10 million was paid in 2020 to support associations alludes to the discussion by Svensson and Radmann [32] that financial support issued by the government for recreational activities was geared toward associations and not commercial actors.

To encourage adherence, in January 2021, a fine of SEK 2000 was introduced for nonadherence to the COVID-19 Act, particularly for gatherings that exceed allowed limits, police could remove members violating congregation restrictions, and those found to be engaging in activities to deliberately spread COVID-19 could be convicted and imprisoned [40]. We must note that nonadherence is, in some cases, due to an inability to correctly interpret restrictions, which has been highlighted by 1 participant. Additionally, it can be a challenging balancing act for the staff concerned to continue to provide recreational activities for youth as is allowed by the Act and maintain social distancing. There were also conflicts with staff ensuring children’s health in a public environment.

Some participants (9/23, 39%) expressed that their municipalities performed infection tracing as necessary, followed routines by Infection Control, and monitored the developments. The highlighted infection tracing pertains to staff and meeting places. During an outbreak, such as COVID-19, more engagement in infection tracing can be expected. It would be interesting to investigate further why only a small number conducted infection tracing, and whether it is a responsibility that lies with another entity.

### Impact and Changes in the Municipal Working Environment

The participants of our study reported that the general impact of COVID-19 in their municipalities constituted closure of recreational activities, limitations and constraints due to the measures in place, and a reduction in activities. According to the majority of the participants (20/23, 87%), many municipalities had to undergo significant changes in their communication, their work processes, and the ways in which they provide services, which was similar to the experience in other countries all over the world. However, a few participants (3/23, 13%) claimed their municipalities were mildly impacted at the time of the survey. We noted that larger municipalities in Sweden (eg, Stockholm and Gothenburg) had higher numbers of COVID-19 infections.

The impact on the working environment constituted changes for staff members and their work conditions, changes in communication, and additional support efforts required for recreational activities. Staff had to adopt different ways of working. For some, responsibilities changed. Moreover, they had to adopt digital alternatives and carry out their work from home, had to change their means of communication to digital forms, and had to provide additional support for associations and recreational activities. For associations, this support was given in the form of information sharing, ensuring there was a common understanding of regulations. Additional effort was required to ensure that continued activities aligned with restrictions and safety measures.

For the general public, the municipalities sought out ways to limit the spread of the COVID-19 virus by ensuring the necessary measures were in place and communicated, but as highlighted by some participants, some exceptions were made for classified members. The responses indicated that some staff still remained and worked in the office. One participant highlighted that in their municipality, administration officials worked from home. This does not imply that everyone worked from home, but rather, it suggests that those who could and needed to work in the office continued to do so. This is further justified by the exceptions that were put in place for some members of the public, such as essential library visits, which would necessitate a worker being present in person.

### Impact of COVID-19 on Recreational Activities

According to the Swedish Local Government Act (Ds 2004:31) [41], municipalities are responsible for recreational and cultural activities in their municipal area. Our results showed that in at least half of the municipalities, recreational activities were closed to the public due to restrictions. However, several other municipalities continued recreational activities with limitations in place, and some municipalities reduced the number and frequency of recreational activities. These findings somewhat contradict some literature contributions, such as the study by Yan et al [3], which stated that “Swedish gyms, schools, restaurants, and shops have all remained open throughout the spread of the pandemic.” However, this may have been true during a certain period of the pandemic. Exceptions were made for children and youth, people with prescriptions, and people using recreational activities for rehabilitation purposes. The concern was that closure of recreational activities can affect public health, especially for vulnerable groups. Similar concerns have been highlighted by Aishworiya and Kang [42], and Deolmi and Pisani [43].

### Efforts Supporting Recreational Activities

The majority of the participants’ municipalities made efforts to keep recreational activities running within the parameters of given restrictions. The guidelines from the public health agency were used as the basis for the provision of recreational activities. Alternatives were also proposed, such as outdoor activities and alternative premises. However, the municipalities had the additional task of having to market the outdoor activities and entice youth to participate in outdoor activities. The latter was found to be challenging. A concern raised in a US study was that low physical activity and sedentary behaviors adopted by children during COVID-19 could be ingrained, increasing the risk of obesity, diabetes, and cardiovascular disease in the future [44]. Conversely, children and youth with an active lifestyle are likely to maintain an active lifestyle in adulthood [45].

### Youth-Focused Support for Leisure Activities

For children, the pandemic presents adverse health outcomes apart from possible infection with the virus. Children with developmental disabilities are more susceptible to the mental health effects of the pandemic, and it is important for their well-being to maintain daily routines, play activities, and recreational activities [42]. In a literature review by Deolmi and Pisani [43], the impact of COVID-19 on the mental well-being of children was addressed, and they found higher levels of stress, anxiety, and depressive symptoms, especially among those who already had chronic diseases. In our study, we found that the restrictions placed on leisure and recreational activities were such that children and youth born in 2002 or later should not be affected. Municipalities made efforts to allow continued access to activities by children and youth. In addition, holiday and cultural activities were organized, which were targeted toward youth, and seasonal staff were employed. Municipalities could differ in implementation.

One participant stated that recreational activities for children and youth not only help maintain physical activity, but also provide safe spaces for those in vulnerable situations. Thus, restrictions cause challenges to municipalities carrying out their core mission and can potentially have more adverse consequences for those in need. This finding is in line with the finding in the report by Deolmi and Pisani [43], who point out that isolation and inability to access recreational activities due to COVID-19 can limit children and youth from having access to emotional support outside the home in cases where they face challenges, such as child abuse, which showed an increase during school closures.

Some participants highlighted closures and limitations affecting access to recreational activities for children and youth despite the Act allowing continuation, highlighting differences among municipalities and between national guidelines and municipal preferences.

The literature covers the importance of engaging in recreational activities, especially by children and youth, and vulnerable groups, and highlights the disadvantages that come with COVID-19 restrictions [21,28-30,42-44]. Sweden and its municipalities have placed priority on the health and well-being of these groups by making efforts to maintain their access to recreational activities even during the pandemic. However, some implementation challenges exist, and some participants referred to closures and limitations that affected children and youth in some areas. Continued physical activity by children, youth, and those with special needs has both short-term and long-term gains [42,45], and Sweden’s approach shows consideration in this regard.

### Role and Significance of ICT

Due to the COVID-19 pandemic, technology use has increased tremendously [46]. Digital platforms were found to support engagement in physical activity for those who had limited access to traditional settings and could not engage in outdoor alternatives in the early months of the pandemic [35]. In Sweden, ICT has played a significant role in municipalities dealing with COVID-19 in several areas.

With restrictions in place, municipalities used technologies that enabled the digitalization of processes, such as the development of digital methods, adoption of technology use for remote work and remote collaboration, expansion of technological solutions, use of social media, and identification of new digital solutions.

Participants expressed that their municipalities used ICT significantly in communication within the municipality and externally. Digital tools became an integral part of communication as people worked remotely during the pandemic, which also demanded continued communication. Digital communication also played a role in maintaining updates and providing reassurance to the public, as well as regularly holding digital meetings with associations.

Municipalities have had opportunities to develop new solutions for use during the pandemic. The new solutions include remote collaboration platforms, and digital solutions for library activities, youth activities, and youth games and quizzes.

The switch to digital technologies during the pandemic in Sweden may have been easier due to Sweden’s openness to digitalization. Digital tool use and online presence were already established to an extent in education, health care, retail, other working environments, and payment systems in Sweden even before the pandemic. Additionally, the internet is accessible to many. Digital tools and internet access contribute to the ease of migration to digital alternatives. However, issues and limitations of existing technologies impose challenges in using them [47].

### Challenges in Using ICT

Though technology adoption has been advantageous, issues have arisen during adoption and use. While some faced issues around privacy and security, and difficulty in complying with the GDPR, others experienced barriers, such as the lack of competence, resources, and licenses. Additionally, it was pointed out that there were challenges in reaching the target group digitally. Digital solutions can also lead to digital isolation for those who lack competence, lack resources, and have disabilities, and for elderly people.

Participants were positive toward the use of ICT in the future; however, new solutions need to be more inclusive for elderly and disabled people. Other countries, such as the United States, which already have digital technologies in place, also see the need to improve digital solutions to better support elderly people, to ensure access to information and services, and to maintain social interaction [46].

### Future ICT Needs and Requirements

The ICT requirements stated by the participating Swedish municipalities highlight a need for development and improvements to facilitate communication, connectivity, efficiency, creativity, better technology, strategic business operations, value creation, and interactivity (Table 2). Further research and assessment of requirements, as well as the development of additional solutions could ensure that the gaps experienced by participants are addressed and that municipalities are better equipped to handle future crises.

### Limitations and Future Work

Due to the COVID-19 crisis, our empirical study had undergone a few design changes. First, since we could not meet our interviewees face to face, we had to adopt online alternatives. However, due to the security issues with using online conferencing tools as well as the scarcity of interviewees, we had to use an online-filled survey. A consequence was that we could not have follow-up discussions on the comments and further investigate the perspectives, since we had mostly 1 participant from each municipality, with the exception of Stockholm, Gothenburg, and Halmstad. However, the format of the online survey allowed us to have responses from the majority of the targeted municipalities, where participants were able to respond at the time of their convenience, and save their progress and return later to complete their responses.

The infection rate and differences among municipalities were not investigated owing to the time of the survey, where infection spread differed and was changing dynamically across municipalities. Future work could investigate the lasting effects of the pandemic in each municipality and whether specific factors of the municipalities or regions, such as size, population, and infection spread, could have affected well-being strategies.

### Conclusions

In this study, we investigated municipal perspectives and approaches to support recreational activities for the public’s well-being. Our study shows that within Sweden’s decentralized government system, municipalities pay attention to guidelines issued at the national and regional levels. Aside from trusting the public with safety guidelines, municipalities showed additional efforts. The ability of municipalities to make their own decisions was noted. Even during the pandemic, they used their right to autonomously govern their jurisdiction to prioritize public health and safety. The results highlight that municipalities were able to regulate the stringency of restrictions issued at the national level when deemed fit. Indeed, Sweden’s approach differed from the approach of several other countries, and it has been criticized for being lenient. However, there is a possibility that what is perceived to be missed at the national level is likely caught at the regional and local levels of governance. The self-rule of municipalities is also evident in the differences between municipal approaches. We appreciate that the differences can be expected to have differing consequences for each municipality. Recreational and leisure activities are the municipality’s responsibility. The need for social distancing resulted in inevitable limitations; however, municipalities offered outdoor alternatives and indirect support through associations. Our results showed that municipal consideration is not limited to the public’s safety against the virus, but extends to the public’s well-being through the support of recreational activities.

The COVID-19 pandemic is a first-of-its-kind event for many, and the lessons learned will equip municipalities with better approaches to manage similar events in the future.

Despite Sweden’s established use of technologies, municipalities still witnessed a notable shift in the ways in which digital solutions are used for work and service provision. Additionally, further requirements are proposed for technology development and process improvement. The important issues highlighted include the need for increased competence and digital inclusion.

## Appendix

Multimedia Appendix 1 Survey questions overview.

Multimedia Appendix 2 English consent form.

## Abbreviations

FOHM: Folkhälsomyndigheten (Public Health Agency of Sweden)
GDPR: General Data Protection Regulation
ICT: information and communications technology

## Data Availability

The data sets generated during or analyzed during this study are available from the corresponding author on reasonable request.
