# Supplementary material for: Handling Public Well-being During the COVID-19 Crisis: Empirical Study With Representatives From Municipalities in Sweden
Source: JMIR Form Res. 2023 May 12;7:e40669. doi: 10.2196/40669 (PMC10185336; doi:10.2196/40669)
Supplement: Multimedia Appendix 1 [file formative_v7i1e40669_app1.pdf]

Q1

1. Which Municipality do you represent?

Other/district (please specify)

Q2

2. What is your position title?

Q3

3. Since COVID19, has there been any significant changes in processes: communication, work, services..etc. within the Municipality?

- No changes
- Minor/ few changes
- Significant changes
- Critical/Major changes
- Don't know

Comment

Q4

4. What is the general impact of COVID19 on recreational activities (visits to museums, gyms, pool, pubs..) in the Municipality?

Q5

5. How important do you think Information and Communications technologies (ICTs) are for dealing with COVID19 restrictions in the Municipality?

- Very significant
- Somewhat significant
- Neutral
- Not significant
- Very insignificant
- Don't know

Comment

Q6

6. Does the Municipality follow any approaches to aid recreational activities during COVID19 (e.g., keep recreational activities ongoing, start new outdoor public activity,..etc.)?

- Yes
- No
- Don't know

Comment

Q7

7. Are there any Municipality specific restrictions are there in place for recreational activities during COVID19?

- Yes
- No
- Don't know

Comment

Q8

8. What measures are taken to ensure safety of COVID19 in the Municipality's recreational businesses?

- National guidelines
- Municipality specific guidelines
- Don't know

Comment

Q9

9. Are there any instances of recreational businesses not adhering to COVID19 restrictions?

- Yes
- No
- Don't know

Comment

Q10

10. Does the Municipality control the adherence to COVID19 restrictions?

- Yes
- No
- Don't know

Comment

Q11

11. Do you have measures for tracking COVID19 cases?

- Yes
- No
- Don't know

Comment

Q12

12. Are there any technologies used in new ways or newly adopted due to COVID19?

- Yes
- No
- Don't know

Comment

Q13

13. Is there a need for new technologies, if so for what purpose?

- Yes
- No
- Don't know

Comment
