# Supplementary material for: Handling Public Well-being During the COVID-19 Crisis: Empirical Study With Representatives From Municipalities in Sweden
Source: JMIR Form Res. 2023 May 12;7:e40669. doi: 10.2196/40669 (PMC10185336; doi:10.2196/40669)
Supplement: Multimedia Appendix 2 [file formative_v7i1e40669_app2.pdf]

Multimedia Appendix 2. English consent form.

With the current pandemic outbreak, guidelines and changes have been implemented on workplaces and business in Sweden. Since recreational activities are important for the overall well-being, we aim to investigate changes and coping mechanisms that businesses have gone through during this crisis. We would like you to help us understand how Covid19 has affected businesses and services in Swedish municipalities.

If you agree to participate, data will be processed according to your informed consent. Participation is completely voluntary and you can withdraw your consent at any time without stating any reason. All data will be used for research purposes only and will be used for academic publications. Data is stored at Karlstad university in an encrypted format and deleted after 10 years. Privacy rules under the EU General Data Protection Regulation (GDPR) will be followed. All will be anonymised and aggregated in our publications. You have the right to revoke your consent at any time during data collection and have the right to request access to your data at any time free of charge. You can request that your data be deleted at any time before the results are published.

Karlstad University is the data controller. According to the General Data Protection Regulation, you have the right to access all information about you collected in this study free of charge and, if necessary, to correct any errors. You also have the right to request deletion, restrict or to object to the processing of personal data, and you can place a complaint with the Data Inspectorate. Contact information for the Data Protection Officer at Karlstad University is dpo@kau.se. For more information on how Karlstad University processes personal data, see https://www.kau.se/en/gdpr.

Contact persons responsible for the study:

Ala Sarah Alaqra [redacted] Senior Lecturer- Information Systems, Karlstad university, as.alaqra@kau.se

Akhona C Khumalo [redacted] Project Assistant - Information Systems, Karlstad university, akhona.khumalo@kau.se
